# Increasing Influenza Vaccination in Primary Healthcare Workers Using Solidary Incentives: Analysis of Efficacy and Costs

**DOI:** 10.3390/vaccines11030557

**Published:** 2023-02-28

**Authors:** Christian Bengoa Terrero, Marian Bas Villalobos, Ana Pastor Rodríguez-Moñino, María Dolores Lasheras Carbajo, Julián Pérez-Villacastín, Cristina Fernández Pérez, María Jesús García Torrent, Rafael Sánchez-del-Hoyo, Alberto García Lledó

**Affiliations:** 1 Servicio de Cardiología, Hospital Clínico San Carlos, 28040 Madrid, Spain; 2 Gerencia Asistencial de Atención Primaria, Servicio Madrileño de Salud, 28035 Madrid, Spain; 3 Dirección General de Salud Pública, Consejería de Sanidad de la Comunidad de Madrid, 28009 Madrid, Spain; 4 Instituto de Investigación Sanitaria del Hospital Clínico, Universitario de Santiago, 15706 Santiago de Compostela, Spain; 5 Departamento de Medicina, Facultad de Medicina, Universidad Complutense de Madrid, 28040 Madrid, Spain; 6 Unidad de Apoyo Metodológico a la Investigación, Hospital Clínico San Carlos, IdISSC, 28040 Madrid, Spain; 7 Servicio de Cardiología, Hospital Príncipe de Asturias, Alcalá de Henares, 28805 Madrid, Spain

**Keywords:** influenza, uptake, healthcare workers, incentives, costs

## Abstract

Introduction: Influenza vaccination campaigns have difficulty in reaching the 75% uptake in healthcare workers (HCWs) that public health organizations target. This study runs a campaign across 42 primary care centers (PCCs) where for every HCW vaccinated against influenza, a polio vaccine is donated through UNICEF for children in developing nations. It also analyses the efficacy and cost of the campaign. Method: This observational prospective non-randomized cohort study was conducted across 262 PCCs and 15.812 HCWs. A total of 42 PCCs were delivered the full campaign, 114 were used as the control group, and 106 were excluded. The vaccine uptake in HCWs within each of those PCCs was registered. The cost analysis assumes that campaign costs remain stable year to year, and the only added cost would be the polio vaccines (0.59€). Results: We found statistically significant differences between both groups. A total of 1423 (59.02%) HCWs got vaccinated in the intervention group and 3768 (55.76%) in the control group OR 1.14, CI 95% (1.04–1.26). In this scenario, each additional HCW vaccinated in the intervention group costs 10.67€. Assuming all 262 PCCs had joined the campaign and reached 59.02% uptake, the cost of running this incentive would have been 5506€. The potential cost of increasing uptake in HCWs by 1% across all PCC (n = 8816) would be 1683€, and across all healthcare providers, 8862€ (n = 83.226). Conclusions: This study reveals that influenza vaccination uptake can be innovative by including solidary incentives and be successful in increasing uptake in HCWs. The cost of running a campaign such as this one is low.

## 1. Introduction

The incidence, morbidity, and mortality of influenza in the population is a challenge across the globe, especially for the elderly and those with at-risk conditions [1,2,3,4,5]. In the United States, the 2017–2018 influenza season was especially severe, with the Center for Disease Control estimating 41 million infections, 21 million medical consultations, 710,000 hospitalizations, as well as 52,000 deaths [6]. In Spain, the numbers are also noteworthy. During the 2011–2012 winter season, excess mortality attributed to influenza was between 8110 and 10,872 [7], and during 2017–2018 even higher, with close to 15,000 estimated deaths [8]. Seasonal influenza vaccination continues to be safe and effective in preventing infection and its large social and economic burden [9,10].

Due to the rapid genetic mutation of the influenza virus, vaccines must be reformulated and re-administered every year. It is the only vaccine that requires annual vaccination in order to offer effective protection against the disease, although it is true that it is currently debated whether the SARS-CoV-2 vaccines will follow suit. Its administration is voluntary and even despite the fact that it is often free, only four of the 38 OECD countries have surpassed 75% uptake in people over 65 years of age at some point over the past three influenza seasons [11].

Even among healthcare workers, who are arguably the most scientifically literate on the value of vaccination, countries rarely achieve a 75% uptake. The World Health Organization, the US Center for Disease Control, and the European Center for Disease Control, as well as most countries, recommend influenza vaccination in healthcare workers; however, reaching high coverage rates remains a challenge [12].

The SARS-CoV-2 pandemic has drastically proven the value of vaccination. It has also revealed that HCWs are particularly vulnerable since they are the first line of defense when outbreaks occur. Its impact on HCW sickness absence is noteworthy; even in a non-pandemic setting, increasing influenza vaccination in HCW by 10% decreases sickness absence rates by 10% in relative terms [13].

In the last few years, and re-fueled by the SARS-CoV-2 pandemic, several healthcare organizations, notably in the United States, have made influenza vaccination mandatory and have been successful in increasing coverage, although not without controversy [14,15,16,17,18,19]. EU countries, including Spain, continue to rely on professional responsibility and targeted campaigns to increase coverage, but uptake was generally below 40% in HCWs before the pandemic [20].

The struggle remains for healthcare systems in reaching their target in HCW vaccination uptake. Public health and employer campaigns are often repetitive and stagnant in their approach and seek new ways to increase uptake each year. To do so, the approach should not be to keep adding evidence to the already vast influenza research but rather to measure specific and driven initiatives as well as their cost. 

The aim of this study was to measure the efficacy and cost of offering a non-monetary incentive to increase uptake in primary care HCWs.

## 2. Materials and Methods

### 2.1. Setting, Study Design, and Population

This observational prospective non-randomized cohort study was conducted across the 262 PCCs (PCC) which belong to the public healthcare network of the Autonomous Community of Madrid (ACM) during the 2020–2021 influenza season. This is a total of 15.812 HCWs assigned to 262 PCCs.

Of the 262 PCCs, 42 belong to a network named CardioRed1, which works alongside four Cardiology Services, one Cardiac Surgery, and one Vascular Surgery Service. CardioRed1 is a clinician-led network to improve cardiovascular health in an area of 1 million people. This project was designed and delivered jointly between cardiology and primary care [21].

The 42 PCCs which belong to CardioRed1 are organically part of four (East, Center, South, and West) of the seven existing primary care districts in the region. These four districts have additional centers which do not belong to CardioRed1. For our study purposes, we defined 3 types of PCCs: those that belong to CardioRed1 (n = 42), those that do not belong to CardioRed1 but organically fall under one of the involved districts (n = 106), and those that are totally separate from CardioRed1 (n = 114).

### 2.2. Implemented Strategies

The 2020–2021 influenza campaign was, at the time, considered to become particularly challenging due to the risk of overlapping influenza and SARS-CoV-2 outbreaks. Knowing this, the researchers reviewed the most successful influenza vaccination strategies and found high uptake rates in England, where 75% of HCWs get vaccinated without mandatory vaccination [22]. 

Two innovative areas of work were identified as a result. Firstly, flu vaccination campaigns aimed at HCWs not only use evidence in their communication strategies but also use amusing and approachable messaging, such as the one found at Liverpool Community Health NHS Trust and others [23]. Secondly, an incentive for vaccination was initially launched by Birmingham Women’s and Children’s NHS Foundation Trust in 2016–2017, where, for every HCW vaccinated against influenza, they donated 10 tetanus vaccines for children and mothers in developing nations through UNICEF [24].

These new approaches were shaped into a ready-to-use campaign for 42 PCCs in the ACM. The sole intervention for the project was producing and sending engaging materials promoting the agreement with UNICEF. In this study, for every HCW vaccinated against the flu, one full polio vaccine (triple dose) was donated to children in developing nations with UNICEF Spain. The list of materials designed and produced can be found in Table 1.

The designed materials were printed and sent to each of the four primary care districts so that they, in turn, would deliver them to their 42 PCCs and, if they chose to do so, other centers within their district.

No other interventions were designed by the research team, and the other elements of the campaign were carried forward as standard across the region.

### 2.3. Defined Groups

Due to the difficulty in the messaging, as well as the inequity in offering the UNICEF incentive to some PCCs of the same district but not others, the four CardioRed1 districts requested that all of their own PCCs be included in the incentive. The research team acknowledged and funded the vaccine for those centers too but did not deliver campaign materials to them, thus creating three distinct groups of PCCs/HCWs:

- Group 1: HCWs who belonged to the 42 PCCs which perceived the full campaign;
- Excluded group: HCWs who belonged to the 106 PCCs, which did not belong to CardioRed1 but did have the UNICEF incentive funded since they were part of the four districts. No special effort was made by the researchers to have the campaign reach them. Due to the risk of contamination, these centers were excluded from this study;
- Group 2: HCWs who belonged to the 114 PCCs that did not belong to CardioRed1. The campaign was unknown to this group.

### 2.4. Data Source and Definitions

The data was provided by the Public Health Directorate of the ACM for the 2020–2021 flu season. Flu vaccination by professional category is offered in bulk and not in relation to each PCC. 

HCWs include all staff involved in healthcare provision, including administrative support, technical staff, etc., and are not limited to frontline clinicians or patient contact.

### 2.5. Statistical Analysis

Both groups were compared with regard to age and sex to assess if there were statistically significant differences between them with the Bonferroni test and chi-squared test, respectively. Data is described as the mean ± standard deviation for age and as a percentage for sex.

The Odds Ratio (OR) was used to compare influenza vaccine uptake among HCWs in the intervention and control groups. Results are shown as OR plus the 95% Confidence Interval (CI). A *p*-value of 0.05 was considered statistically significant. 

For this purpose, the Software package IBM SPSS Statistics version 22 was used (IBM Corp, Armonk, New York, NY, USA). 

### 2.6. Cost Analysis

UNICEF Spain offers 135 polio vaccine doses for 26.45€, making each dose 0.1959€. Since a triple dose is necessary for full protection, funding a complete polio vaccine costs 0.59€ per HCW vaccinated against influenza (cost data from 2021) [25]. UNICEF is likely to have other costs to reach children with the polio vaccine, which are not included in the incentive.

If a hospital, region, or public health team were to deliver a similar campaign, the printing and delivery of the materials would stay stable since it is a recurring cost each year. Adapting the campaign would not increase its budget since it’s rebranded yearly too. However, financing the polio vaccine would incur an additional cost which is why the analysis only includes the additional financing of the incentive. 

We have also structured four scenarios by changing potential uptake either across all PCC or across all healthcare providers, i.e., including hospital and ambulance providers. 

Additionally, we have calculated the cost of a 1% increase by dividing the potential cost of running the campaign across all PCCs and the potential increase in uptake from running it.

## 3. Results

### 3.1. Healthcare Worker Vaccination Uptake

We found no statistically significant differences between Group 1 and Group 2 in age (46 ± 13.1 vs. 45.8 ± 13.3, *p* > 0.999) or sex (female 79.7% vs. 79%, *p* = 0.514).

Table 2 simplifies into 13 categories the 46 different roles and contractual arrangements of employment available in the PCCs included in the evaluative study. This table includes a larger sample of 20,408 since it also includes staff who work for primary care but not for a specific center. Among others, those additional roles primarily include management, facilities, estate teams, public health, information technology technicians, as well as clinical technicians. 

### 3.2. Efficacy

Flu vaccination uptake for the 262 PCCs and 15,812 HCWs for the winter season of 2020–2021 was 56.61%. The highest uptake in a single center was 80%, and the lowest uptake was 20%. The average number of HCWs per PCC is 60 (median = 58). The largest PCC employs 157 workers, and the least, 13. No correlation was found between the number of staff employed and percentage uptake in HCWs (r = −0.05). Our data also shows 68 of the 262 PCC were ≤50%, and 13 PCCs were ≤40% uptake in HCWs.

Group 1, which was subject to the campaign, with 2.411 HCWs distributed across 42 centers, achieved 59.02% uptake. Group 2, which was unaware of the campaign, had an uptake of 55.76% in 6758 HCWs (114 PCCs), (Table 3). 

An odds ratio with a 95% confidence interval shows that belonging to Group 1 increases the likelihood of vaccination by 14% OR 1,14, CI 95% (1.04–1.26) when compared to Group 2. 

### 3.3. Cost Analysis

As agreed by the research team with the primary care districts, the incentive for HCWs vaccinated in Group 1 was financed, resulting in a cost of 840€.

Had it been attempted, the additional cost of delivering the campaign with the UNICEF incentive across the whole of primary care in the region would have cost 5506€. This assumes all 15,812 PCC would have reached the same uptake in HCWs as Group 1 (59.02%), which would have resulted in a total of 9332 staff vaccinated against influenza. If this had been attempted across all public healthcare providers in ACM, including hospital and primary care (n = 83,226), still assuming a 59.02% uptake, it would have cost 28,980€. If the whole region eventually reached 75%, it would cost a total of 36,827€. Table 4 summarizes these scenarios. 

To assess the cost per additional vaccinated HCW, a series of considerations must be made. Group 1 had a 3.27% higher flu vaccination uptake than Group 2. Assuming the campaign achieved equal influenza vaccine coverage across the whole region (59.02%), this totals an additional 516 HCWs vaccinated against influenza in primary healthcare centers.

In this simulated scenario, delivering the campaign would have cost a total of 5506€ while achieving 516 additional vaccinated HCWs. This equates to an additional 10.67€ per additional HCW vaccinated against influenza.

The data in Table 4 and Table 5 allow for the calculation of the cost of a 1% increase. The potential cost of increasing uptake in HCWs by 1% across all PCCs would be 1683€ (8816/3.27), and across all healthcare providers, 8862€ (83,226/3.27).

## 4. Discussion

Our study demonstrates that a campaign offering solidary incentives for influenza vaccination can increase vaccine uptake among HCWs even in such a complex scenario as the SARS-CoV-2 pandemic. Influenza vaccination for the 2020–2021 season reached 56.4% in our sample but, compared to the previous year, went up from 36.6% in HCWs in the ACM [26]. This increase in uptake can only be explained by the SARS-CoV-2 pandemic, which heightened the importance of vaccination both in the general public and in HCWs. This is a worldwide trend [11,27,28,29,30,31,32] that has probably had an impact on the efficacy of the campaign since most staff did not need an additional motive for vaccination. Nonetheless, our results still reveal a significant difference in a large sample size between the two distinct groups. In a non-pandemic setting, the effect of the campaign would have most likely been greater. 

Further confirmation that our campaign is the cause of this variation comes from the excluded group. As mentioned previously, these PCCs were excluded because of the possibility of becoming aware of the campaign, arguably less so than the intervention group but more so than the control group. The excluded group lies precisely between both groups as evidence that some permeability is likely to have occurred. 

Had there been a larger disparity between the groups, the cost per additional vaccinated HCW would be below the calculated 10.67€. However, considering the substantial economic burden of absenteeism during influenza epidemic periods, it will likely be quickly offset. In a four-site Italian hospital study, the authors elaborately calculated extra days lost due to flu (EDLF) and its cost from lost work days. From its sample of 5401 HCWs, this reached a cost of 1,763,683€ across three influenza seasons [33]. While this conclusion should be accepted with caution, it reveals the large burden of HCW sickness absence for health systems.

While it is not focused on cost, the largest study on the impact of influenza vaccination on sickness absence was carried out in the NHS in England with data from 800,000 HCWs and 245 NHS Trusts (i.e., large healthcare provider organizations). They analyzed the relationship between sickness absence rates and influenza vaccine uptake. Vaccine uptake was significantly associated with a reduced sickness absence rate (β = −0.425, *p* < 0.001). In particular, an increase of 10% in influenza vaccine uptake was able to reduce 0.43% in the absolute sickness absence rate. Considering that the average sickness absence rate was 4.5% across the four influenza seasons, this translates into a 10% relative decrease in the sickness rate [13]. A recent meta-analysis of the impact of vaccination on sickness absence confirms it is cost-effective to vaccinate HCWs. However, for patient nosocomial infection from HCWs, it concludes that little research is available specifically on how HCW vaccination protects patients and is eventually cost-effective [34]. Overall, most cost-effectiveness studies and reviews argue in favor of influenza vaccination in patients and HCWs [35,36,37].

A solidary incentive costing 0.59€ per vaccinated HCW seems appropriate and can increase the perception of the benefit of vaccines among HCWs and the general public. If it were performed across a whole region or country, it would certainly grasp social interest, and, therefore, its impact would be greater, making it more worthwhile.

The relatively small cost of increasing uptake by 1% in such a large sample of HCWs truly shows the potential this could hold. However, its increase would likely not be linear as more resistant HCWs will undoubtedly remain regardless of the incentive. 

Additionally, the reach and influence of the research group were more limited than if it had been a centrally driven campaign. It could be argued that the differences found here would be increased without much additional effort from a central public health team.

Only eight primary care health centers reached or surpassed 75% vaccination uptake in HCWs demonstrating that much work still needed to be performed to increase uptake. Our data also revealed a 60% gap between the highest and lowest PCC uptake. Some learning could be transferred from top-performing PCCs to the rest, and some driven interventions could be made to the bottom end. This is further reinforced by how 68 of the 262 PCC were equal to or below 50%, and 13 PCCs were equal to or below a 40% uptake. Increasing uptake in low-uptake PCCs is most likely less challenging since the margin for improvement is larger. 

The ACM is below average in HCW influenza coverage when compared to the rest of the country, which reached 65.7% [26]. It is important to note that other countries have a better definition of at-risk healthcare workers, restricting it to frontline roles, which in all probability favor their results.

The results reveal that nursing and medical staff have the highest uptake. Other HCWs in close contact with patients, such as physiotherapists and nursing assistants, have a very low uptake, so specific efforts must be made to target these specific groups.

### Limitations

This is not an experimental study, and, therefore, no controlling factors were addressed beforehand; nonetheless, it can be assumed that the samples are homogenous—they belong to the same health system and provide the same services under the same contractual arrangement, in a small territory within a geographically contained area and where the influenza campaign is identical across the control group. Additionally, a group of PCCs was excluded, which could have been partially influenced by the campaign. It turns out that this excluded group precisely lies between the control and intervention groups and serves as further confirmation that the campaign had an impact. Furthermore, there are no statistical differences in either age or sex between the studied groups.

Additionally, it could not be determined whether it was the UNICEF incentive or the other materials that encompassed the campaign which had the effect on increasing uptake. The results shown here only truly demonstrate the effectiveness of the campaign as a whole. It would be of added value to introduce a qualitative survey that addresses the motivations behind influenza vaccination in HCWs. Future studies should consider introducing this so as to discern the real impact of the solidary incentive.

## 5. Conclusions

A comprehensive campaign, which includes innovative materials and a solidary incentive, has a significant impact on flu vaccination uptake in HCWs. Even considering that the impact was probably softened by the heightened awareness of vaccines due to the SARS-CoV-2 pandemic, the cost analysis reveals that a campaign of this nature is inexpensive and could be used for local, regional, or national public health campaigns. As far as we are aware, this is the only study to quantify on this scale the cost and effectiveness of a solidary incentive. This incentive is not a silver bullet and must be applied with other evidence from vaccination programs, for example, by including messaging on the safety of influenza vaccination, etc. [38]. The learning from our study could be extrapolated to SARS-CoV-2 vaccination campaigns, especially if they were to become annual or combined with the influenza vaccine. We encourage other health systems to implement this incentive and publish their results.

## Figures and Tables

**Table 1:** Materials, messaging, and quantities sent to the primary care districts.

Item	Messaging	Amount Printed
A4 posters in color	“Getting vaccinated makes you more attractive and prevents flu * * Only 50% of this statement is supported by evidence.”	1136
	“It’s time to get vaccinated against flu. Yes, healthcare staff too.”	
	“A nurse vaccinated against flu transforms into a child vaccinated against polio.”	
	“There is no vaccine against COVID-19, but there is against flu.”	
	“We promise it’s the last time you get vaccinated against flu. (until next year of course).”	
Jab-o-meter	A 2-m roll-up banner of a vaccine where percentage uptake is filled in with a marker.	42
Stickers	Circular stickers for vaccinated staff so that patients can read them on their badges “Vaccinated against flu to protect you 2022.”	230 sheets of stickers with 70 stickers per sheet

* All materials produced included UNICEF’s logo as well as the message “CardioRed1 will donate one vaccine against polio for every healthcare worker vaccinated against flu”.

**Table 2:** Flu vaccination uptake in primary care by role.

Role	Number of HCW	Vaccinated HCWs	Percentage Uptake
Pediatricians	899	643	71.5
Resident doctors	922	588	69.4
Doctors	4724	3008	63.4
Matrons	319	197	61.8
Administrative staff	4024	1922	59.9
Nursing staff	6017	3475	59.2
Others	1227	584	58.6
Oral health clinicians	237	117	49.4
Social workers	139	65	46.8
Dentists	229	100	43.6
Nursing assistants	1200	610	42.2
Physiotherapist	471	197	41.8
Total	20,408	11,506	56.4

**Table 3:** Influenza vaccination rates in HCWs by group.

	HCW	Vaccinated HCW	Uptake (%)
Group 1: HCWs belonging to the 42 PCCs which received the full campaign	2411	1423	59.02 *
Group 2: HCWs of the 114 PPCs that did not belong to CardioRed1	6758	3768	55.76
Excluded group (HCWs from 106 PPCs)	6643	3761	56.62
Total:	15,812	8952	56.62

* *p* < 0.001 for the comparison between group 1 and group 2.

**Table 4:** Cost of funding the UNICEF incentive in four simulated scenarios.

	Assuming 59.02% Uptake	Assuming 75% Uptake
All public PCCs HCWs (n = 15.812)	5506€	6997€
All HCWs employed in a public healthcare provider. * (n = 83,226)	28,981€	36,827€

PCC: Primary care center. HCW: Healthcare worker. * Includes HCWs from all public healthcare providers of the ACM: primary care, hospital care, and the public emergency ambulance provider.

**Table 5:** Comparison in uptake and cost if the campaign had been delivered across the whole region versus not at all.

If No Campaign Had Taken Place (Based on Data from Group 2)			If the Campaign Has Been Delivered across the Whole Region (Based on Data from Group 1)			Difference	
n	%	€	n	%	€	n	%
8816	55.76	0	9332	59.02%	5506	516	3.27
